# Regulation of Cardiac PKA Signaling by cAMP and Oxidants

**DOI:** 10.3390/antiox10050663

**Published:** 2021-04-24

**Authors:** Friederike Cuello, Friedrich W. Herberg, Konstantina Stathopoulou, Philipp Henning, Simon Diering

**Affiliations:** 1 Institute of Experimental Pharmacology and Toxicology, Cardiovascular Research Center, University Medical Center Hamburg-Eppendorf, Martinistrasse 52, 20246 Hamburg, Germany; k.stathopoulou@uke.de (K.S.); saimoon@hotmail.de (S.D.); 2 DZHK (German Center for Cardiovascular Research), Partner site Hamburg/Kiel/Lübeck, University Medical Center Hamburg-Eppendorf, Martinistr. 52, 20246 Hamburg, Germany; 3 Department of Biochemistry, University Kassel, Heinrich-Plett-Str. 40, 34132 Kassel, Germany; P.Henning@uni-kassel.de

**Keywords:** kinase, phosphatase, cardiac myocyte, contractile function, oxidation

## Abstract

Pathologies, such as cancer, inflammatory and cardiac diseases are commonly associated with long-term increased production and release of reactive oxygen species referred to as oxidative stress. Thereby, protein oxidation conveys protein dysfunction and contributes to disease progression. Importantly, trials to scavenge oxidants by systemic antioxidant therapy failed. This observation supports the notion that oxidants are indispensable physiological signaling molecules that induce oxidative post-translational modifications in target proteins. In cardiac myocytes, the main driver of cardiac contractility is the activation of the β-adrenoceptor-signaling cascade leading to increased cellular cAMP production and activation of its main effector, the cAMP-dependent protein kinase (PKA). PKA-mediated phosphorylation of substrate proteins that are involved in excitation-contraction coupling are responsible for the observed positive inotropic and lusitropic effects. PKA-actions are counteracted by cellular protein phosphatases (PP) that dephosphorylate substrate proteins and thus allow the termination of PKA-signaling. Both, kinase and phosphatase are redox-sensitive and susceptible to oxidation on critical cysteine residues. Thereby, oxidation of the regulatory PKA and PP subunits is considered to regulate subcellular kinase and phosphatase localization, while intradisulfide formation of the catalytic subunits negatively impacts on catalytic activity with direct consequences on substrate (de)phosphorylation and cardiac contractile function. This review article attempts to incorporate the current perception of the functionally relevant regulation of cardiac contractility by classical cAMP-dependent signaling with the contribution of oxidant modification.

## 1. Introduction

**Figure 1:**
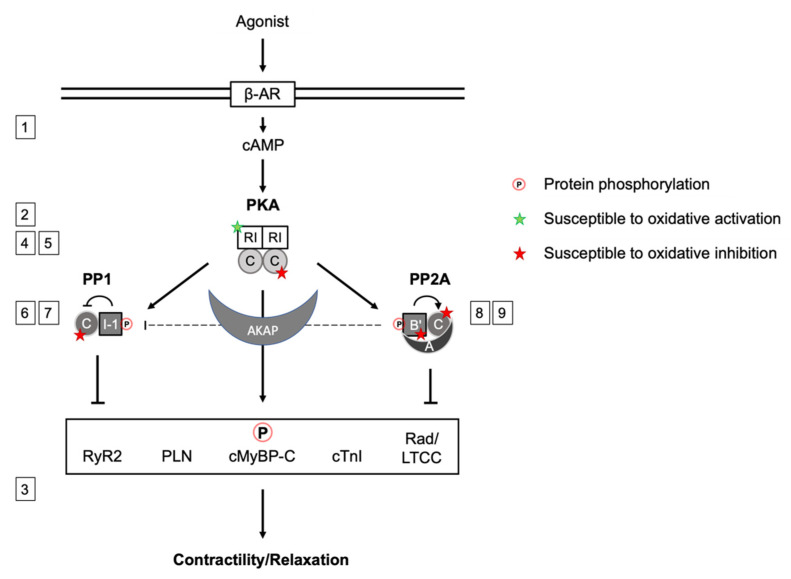
Scheme summarizing the signaling network between PKA, PP1 and PP2A, their inter-action upon βAR stimulation and cAMP production or direct oxidative modification. PKA phosphorylates and activates inhibitor 1 (I-1), which inhibits PP1. Several B’-PP2A regulatory subunits have been shown to be subject to phosphorylation, B56δ is phosphorylated by PKA, enhancing PP2A activity by dissociation from the A-subunit. PP2A mediates I-1 dephosphorylation. PKA phosphorylates substrate proteins in cardiac myocytes that regulate cardiac contraction, which is counteracted by PP2A and PP1 action. In addition to the regulation by phosphorylation, signaling subunits labeled with a star are susceptible to oxidation. Green: enhanced activity, red: inhibitory effect. The scheme synergizes aspects of the regulation of PKA, PP1 and PP2A activity by phosphorylation and simultaneous oxidation to orchestrate contractility. Numbers 1–9 referring to the respective section in the manuscript.

The sympathetic nervous system represents the most powerful regulator of cardiac contractile function. Thereby, the monoamine neurotransmitters epinephrine and norepinephrine innervate postsynaptic β-adrenoceptors on the surface of cardiac myocytes allowing canonical intracellular signal transduction. This involves the generation of the intracellular second messenger 3′-5′-cyclic adenosine monophosphate (cAMP) and subsequent activation of its principle cellular effector enzyme cAMP-dependent protein kinase (PKA). Incessant investigations are focusing on understanding the isoform-specific, spatiotemporal and structural contributions as well as disease-specific alterations in PKA signaling. PKA is “the kinase” in cardiac myocytes that links neurohumoral extracellular signals to changes in contractile function. This process termed excitation-contraction coupling occurs via phosphorylation of key substrate proteins [1,2] (Figure 1).

## 2. PKA Isoforms

PKA is a heterotetrameric enzyme that is composed of a dimeric regulatory (R) and two catalytic subunits. Several PKA-C isoforms are expressed in human tissue: Cα, Cβ and PrKX. Cβ isoform expression appears to be predominantly in the nervous and the immune system and PKA-Cα is ubiquitously expressed [3,4]. In humans, *PRKACG* (Cγ) and PrKY have been described as retrotransposons [5,6]. Association of PKA-C with either of the two existing types of regulatory subunits, RI or RII, defines the holoenzyme as type I or type II PKA. While the expression of RI and RII differs substantially between tissues, type I PKA activity predominates in the heart [7]. Importantly, attempts to quantify PKA subunit abundance in the heart, revealed high abundance of RI subunits with an approximately 10-fold [8] or even 17-fold [9] excess over the catalytic subunits. This observation was rationally explained by RI functioning as a cAMP storage allowing the formation of biomolecular condensates enriched in cAMP and PKA activity, critical for effective cAMP compartmentation [10]. Also, the concept of “buffered cAMP diffusion” supports the biological significance of RI abundance: cAMP at physiological concentrations is predominantly bound to cAMP binding sites and thus immobile [11]. Those sites could potentially be RI subunits.

For both, RI and RII regulatory subunits, α and β isoforms have been described [12]. Generally, α isoforms are expressed ubiquitously and β isoforms display enhanced tissue specificity. As PKA-RIβ transcripts are detected in brain and testis, but not in the heart [13,14], it is assumed that PKA-RIα prevails in cardiac tissue [4,15]. Control of physiolo-gical effects in cardiac tissues is mediated via A-kinase anchoring proteins (AKAPs) predominantly interacting with type II R-subunits and regulating Ca^2+^ release and uptake [16]. All regulatory subunit isoforms share the same domain structure. At the N-terminus, a dimerization and docking (D/D) domain that mediates PKA-R dimerization and interaction with AKAPs is followed by a pseudo-substrate inhibitory sequence, which in the absence of cAMP reaches into the active site cleft of the catalytic subunit [17]. The two large C-terminal cAMP-binding domains account for approximately two thirds of the subunit [18,19] allowing for the cooperative activation of the respective holoenzymes [20].

## 3. Cardiac PKA Substrate Phosphorylation and Contractile Function

PKA substrates in cardiac myocytes that regulate contractile function involve both, myofilament proteins and those involved in intracellular Ca^2+^-cycling. PKA-mediated phosphorylation of the Ca^2+^ channel inhibitor Rad (Ser25/38/272/300), a Ras-like GTP-binding protein family member, relieves its constitutive inhibition on Ca_V_1.2. This increases channel open probability and thus contributes to the inotropic response [21]. At the sarcoplamic reticulum (SR), the action of PKA promotes both, the import of Ca^2+^ into the SR and its release into the cytosol by the ryanodine receptor (RyR2). PKA-mediated phosphorylation of SR membrane-associated phospholamban (PLN) at Ser16 abolishes its inhibitory effect on the sarcoplasmic/endoplasmic reticulum Ca^2+^-ATPase (SERCA2), resulting in accelerated Ca^2+^ import into the SR during diastole and, in consequence, positive lusitropy [22,23,24]. At the same time, phosphorylation of RyR2 by PKA promotes the release of Ca^2+^ during systole and enhances contractility [25]. Within the myofilament contractile machinery, PKA-mediated phosphorylation of cardiac myosin-binding protein C (cMyBP-C) at three sites of its cardiac-specific M-motif releases the constraint of cMyBP-C on myosin S2 head domains. This enforces actin-myosin interaction and acceleration of cross-bridge cycling, thus contributing to the positive inotropic effect [26,27]. An important feature of increased PKA activity in cardiac myocytes is the reduction in sarcomere Ca^2+^ sensitivity that has been attributed to direct myofilament protein phosphorylation. Thereby, Ca^2+^ desensitization of the myofilaments is reduced by PKA-mediated phosphorylation of cardiac troponin I (cTnI) at Ser23/24, a component of the heterotrimeric troponin complex [28,29,30] and cMyBP-C, providing improved sarcomere relaxation [31,32,33]. Phosphorylation of cTnI lowers the affinity of the cTnC subunit for Ca^2+^, further enhancing the PKA effect on sarcomere Ca^2+^ sensitivity and cardiac lusitropy [34,35,36].

## 4. Oxidative Post-Translational Modification of PKA-RI

It is commonly accepted that PKA represents the main effector for intracellular cAMP and that binding of the second messenger activates the kinase. Interestingly, already de-cades ago in the 1980s, researchers described oxidation of PKA. However, only recently increasing research efforts were undertaken to pinpoint the nature and functional importance of PKA oxidation on specific cysteinyl thiols. This timely delay might be due to the former lack of availability of sophisticated knock-in animal models and techniques to study protein oxidation in complex homogenates [37]. Furthermore, there is increasing perception that oxidative post-translational modifications resulting from controlled gene-ration of reactive oxygen species (ROS) on key proteins plays an important physiological role in the regulation of fundamental processes such as angiogenesis, vascular tone or cardiac contractility.

Oxidative post-translational modification of type I PKA regulatory subunit (RI) was first described by Potter and Taylor in 1980 [38], who investigated PKA-RI and its proteolytic fragments in porcine skeletal muscle. The observation of bands migrating at higher molecular weight during native SDS-PAGE analyses that were exclusively detectable in the absence of the reducing agent β-mercaptoethanol, gave rise to the hypothesis of N-terminal sulfhydryl groups forming an interdisulfide-linked PKA-RI dimer [38]. This fin-ding represented a significant discovery, as it had been considered a “general rule” that oxidative disulfide bonds were exclusively found in extracellular proteins and their formation prevented by the reducing intracellular environment. Thus, “the occurrence of disulfide bonding in an intracellular protein” was perceived as “highly unusual” [39]. Further studies on PKA-RI, however, could localize two oxidized cysteine residues to a small N-terminal fragment of 5400 Da. This eventually identified Cys17 and Cys38 (referring to rat sequence) as the amino acids involved in the two antiparallel interdisulfide bonds that can form between two PKA-RI monomers [39,40,41]. PKA-RII did not display any reducing agent-mediated differences in its migration pattern upon SDS-PAGE experiments. This can be rationally explained by the lack of both cysteines, Cys17 and Cys38 [39]. Another remarkable feature that was characteristic for this bond that crosslinks two PKA-RI protomers was its high stability. Treatment with 30 mM of the reducing agent dithiothreitol (DTT) was insufficient to reduce the bond [39] and even pretreatment with 8 M urea did not lower the concentration of DTT required to recover the monomeric form [41]. More-over, although located within the dimerization and docking domain, Cys17-Cys38 interdisulfide bond formation was not required for the dimerization of PKA-RI, an essential prerequisite for the formation of the heterotetrameric structure of the holoenzyme [41]. At this point in time, it was uncertain, though, whether the PKA-RI interdisulfide was a constitutive structural feature, a purification artefact or in fact an inducible post-translational modification with physiological functional relevance.

An important advancement to answer this important issue was provided by the work of Brennan et al. in 2004, who identified PKA-RIα among the proteins that underwent oxidative dimerization in response to treatment with the disulfide-inducing agent diamide in rat ventricular cardiac myocytes by unbiased two-dimensional SDS-PAGE [42]. This finding provided evidence that oxidative PKA-RI dimerization was indeed an indu-cible event occurring in intact cardiac myocytes and strongly suggested that this modification may have functional consequences concerning cellular PKA actions. Shortly after, the same group reported elevated phosphorylation levels of PKA substrate proteins following incubation of cardiac myocytes with the exogenous oxidant H_2_O_2_ [43]. Protein phosphorylation was paralleled by interdisulfide PKA-RI dimer formation, but was attenuated by the inhibitor of PKA-C, H89, while cAMP levels remained unaltered after exposure to H_2_O_2_. These results suggested the following order of events: oxidant-mediated disulfide formation in RI releases PKA-C, without the necessity of prior cAMP-binding. Altogether, these data provided solid indication for oxidative PKA-RI dimerization as a novel activatory mechanism of PKA. Thereby, activation might occur independently from classical catecholamine-evoked β-AR signaling with impact on phosphorylation-regulated cardiac contractility.

A recent study performed by our group partially supported the above-described observations, but pointed to a more complex scenario regarding the functional outcome of PKA oxidation in a cardiac myocyte context. When comparing the impact of an array of model oxidants on PKA oxidation and cardiac myocyte function [44], we observed a disconnect between interdisulfide formation in PKA-RI and substrate phosphorylation. Diamide as well as 1-nitrosocyclohexyl acetate (NCA), a donor of the small molecule HNO that has raised great interest as a potential treatment for acute decompensated heart failure, induced pronounced PKA-RI dimer formation. This was paralleled by phosphorylation of the PKA substrate proteins cMyBP-C located at the myofilaments and the SR/ER-associated PLN. FRET experiments using an A-kinase activity reporter confirmed these phosphorylation events to rely on increased PKA-C activity. Exposure to the β_1_-AR anta-gonist atenolol suggested that the effects occurred independently from the classical receptor-mediated activation pathway. Surprisingly, oxidative PKA-RI dimer formation was also detectable after exposure to H_2_O_2_, which failed to evoke detectable effects on protein phosphorylation in our experiments. All tested compounds resulted in reducing agent-reversible disulfide crosslinking of PKA-RI subunits and highlighted that in cardiac myocytes oxidative PKA-RI dimerization alone was not sufficient to activate the kinase. It is unlikely that exposure of cardiac myocytes to different oxidants could result in the formation of different kind of disulfide bonds between PKA-RI protomers with a potentially differential impact on kinase activity. Previous work had convincingly determined the orientation of PKA-RI protomers within the dimer, supporting the conception of the antiparallel Cys17-Cys38 bond [40,41]. Moreover, the involvement of additional cysteine re-sidues that exist within the PKA-RI cAMP-binding sites had been excluded [38,39]. Activation of PKA independently of cAMP could potentially involve additional oxidation events apart from RI interdisulfide linkage that remain to be identified and characterized in future studies. Interestingly, for RIβ and RIIβ even “a dimer of a dimer” structure was convincingly postulated: A tetrameric structure composed of two RIIβ(2):C(2) holoenzymes was described that provided mechanistic insight into the allosteric activation by cAMP [45]; quaternary structures of RIβ selectively enriched in the mitochondria forming “discrete signaling foci” [46].

A valuable tool in the delineation of the physiological consequences of PKA-RI interdisulfide formation in vivo represented the PKA-RIα Cys17Ser knock-in mouse model [47]. “Redox dead” mice replacing Cys17 by non-oxidizable serine revealed defective angiogenesis upon hind limb ischemic injury. Interestingly, a recently published study described no impact of the disulfide dimer on PKA catalytic activity, but instead an increased binding affinity to AKAPs with functionally important subcellular redistribution attenuating injury following ischemia/reperfusion [48]. These data are in accordance with our study that rather than impacting directly on kinase activity, emphasized an important role of interdisulfide formation in PKA-RI for oxidant-mediated translocation into substrate vicinity. In the atria, PKA-RI dimer formation upon oxidant-exposure has been described to contribute to the prolongation of cardiac action potential duration and occurrence of after depolarizations due to inhibition of *I*_to_ and *I*_K1_ [49]. Although the authors described increased oxidant-mediated disulfide formation leading to increased PKA activity, they did not assess kinase activity. Therefore, it is likely that the effect on ion channel activity that they observed resulted from increased kinase translocation into channel vicinity. Alignment of the amino acid sequence of PKA-RI reveals that mouse PKA-RI contains an additional cysteine residue at position 66 that is absent in the human and rat sequence. This additional cysteine residue might be susceptible to oxidation as well and might be alternatively involved in disulfide bond formation with Cys38, potentially compensating for Cys17, which appears structurally possible. Thus, future experiments should be performed in human cardiac myocytes to verify a definitive correlation between PKA-RI dimer formation and kinase activation.

**Figure 2:**
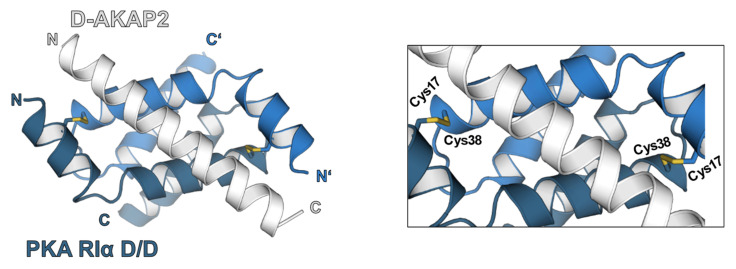
Structure of PKA-RIα dimerization and docking domain (D/D-domain) in complex with D-AKAP2 [52] (PDB 3IM4)**.** The RIα D/D-domain (blue and dark blue) is defined by an N-terminal helical bundle that promotes regulatory subunit dimerization via hydrophobic interactions. Under oxidative conditions, additional intermolecular disulfide bonds are formed by cysteines at position 17 and 38. The interaction with different AKAPs (grey) is mostly caused by hydrophobic interactions alongside salt bridges. An effect of the interdisulfide bond formation on D/D-domain-AKAP interaction and PKA localization could be shown by different studies [43,48].

Enhanced PKA-RI-AKAP interaction might be the key function of oxidant-mediated PKA-RI disulfide formation and might fine tune or alter the substrate spectrum of the kinase by impacting on kinase compartmentation during conditions of increased oxidant production. This observation was reported early onwards by Brennan at al. [43], when demonstrating the translocation of oxidized PKA-RI into the myofilament-containing subcellular fraction of H_2_O_2_-exposed cardiac myocytes. This allowed to formulate the hypo-thesis that the interdisulfide bond may alter the affinity of PKA-RI for certain AKAPs that guide oxidant-mediated PKA activity to particular cellular compartments. This assumption was indeed well founded, as the N-terminal D/D-domain of PKA-R that in the type I isoform harbors Cys17 and Cys38 does not only mediate regulatory subunit dimerization, but also the interaction with AKAPs (Figure 2). In line with these results, the data of various studies showed subcellular translocation of PKA into the myofilament containing fraction of adult rat ventricular myocytes after exposure to NCA [44], to the lysosomal two pore channel during ischemia/reperfusion in the ventricle [48] or into cation channel vicinity to trigger arrhythmias upon oxidant-exposure [49]. A potential impact of PKA-RI disulfide formation on the interaction with AKAPs had previously been investigated for D-AKAP1, the first AKAP that was found to not exclusively bind to type II, but to be dual-specific and also interact with type I PKA regulatory subunits [50]. For the interaction with this particular anchoring protein, however, dependence on RI oxidation could not be confirmed, as Cys38, but not Cys17 was found to be essential for the interaction of kinase and D-AKAP1 [51].

**Figure 3:**
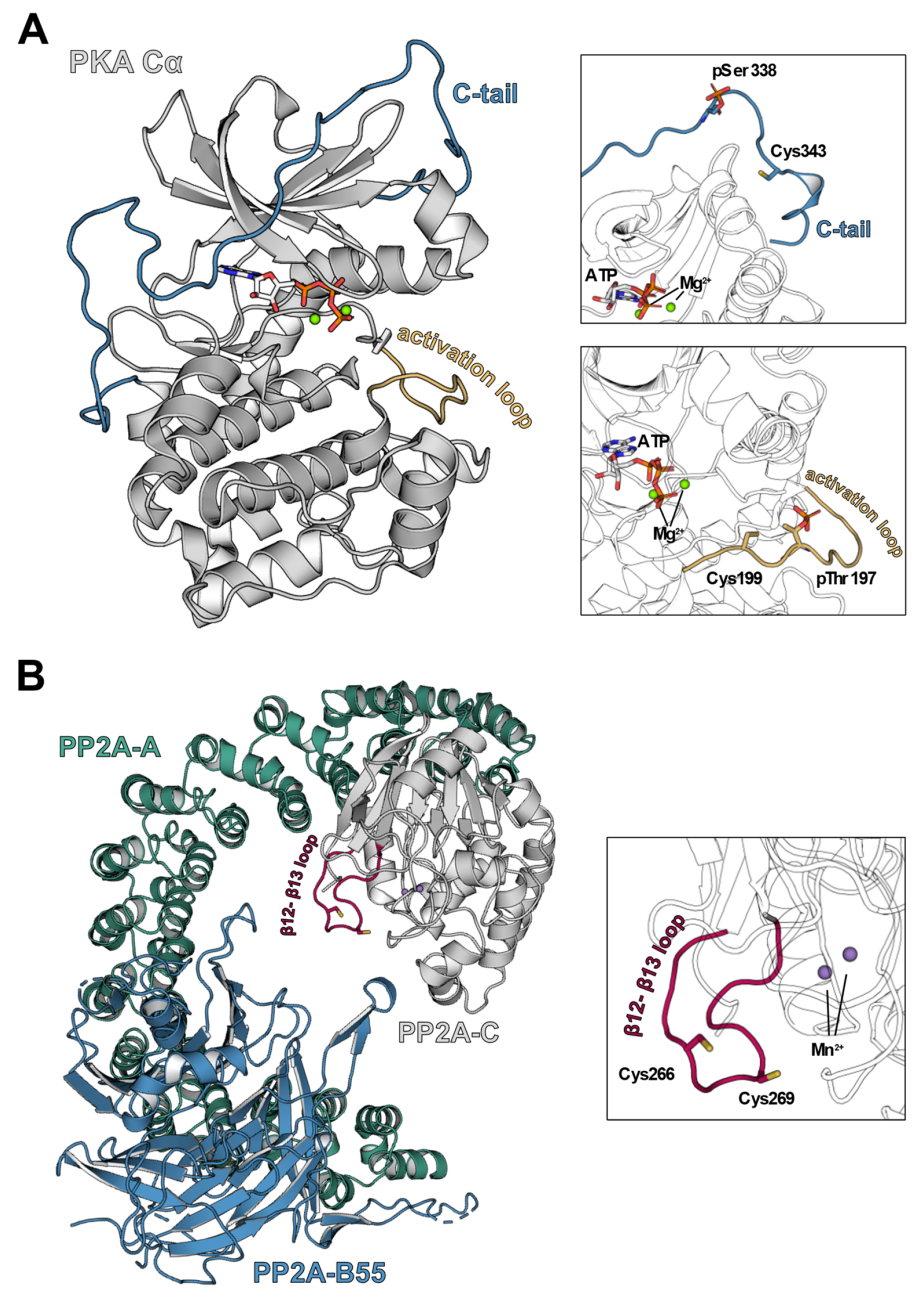
Structures of PKA-Cα and PP2A in ribbon representation. (**A**) cAMP-dependent protein kinase catalytic subunit Table 1. ions [60] (PDB 4WB5). The catalytic subunit of PKA consists of two lobes, the N-lobe consists mainly of β-sheets and the C-lobe comprises α-helical structures. In the latter, a redox-sensitive cysteine, Cys199, is located in the activation loop (yellow) close to the catalytic cleft, which was shown to inhibit kinase activity upon oxidation. Another cysteine susceptible to redox processes, Cys343, is found in the C-terminal tail (C-tail, blue) that spans from the C-lobe to the N-lobe of PKA-Cα. (**B**) Hetero trimeric holoenzyme of protein phosphatase 2A (PP2A) [61] (PDB 3DW8). The fully active PP2A holoenzyme consists of the core enzyme comprising the scaffold unit PP2A-A (teal) and the enzymatically active subunit PP2A-C (grey) as well as a regulatory subunit, here PP2A-B55 (blue). Localized on the β12-β13 loop of PP2A-C (magenta), there are two redox-sensitive cysteines, Cys266 and Cys269, that contribute to an oxidation-induced inhibition of PP2A-C via disulfide bridge formation.

An important function of the D-AKAP1-PKA interaction is to ascertain mitochondrial localization of PKA in neuronal cells [53]. Here, PKA-mediated phosphorylation of the dynamin-related protein (Drp1) at Ser637 regulates mitochondrial fission and function [54,55]. A decrease in neuroprotective PKA signaling is associated with the development of neurodegenerative diseases and excessive ROS production. Several other AKAPs may potentially anchor the PKA holoenzyme to sarcomeric substrates as a result of an altered affinity for oxidized PKA-RI. Myosin heavy chain, which is directly associated with one of the main targets of phosphorylation in the sarcomere, namely cMyBP-C, has been suggested as a novel interaction partner of disulfide-linked PKA-RI [43]. Such interaction would anchor PKA in the immediate proximity to its substrate at the thick filament and would explain translocation and myofilament protein phosphorylation upon oxidant-exposure. Equally plausible would be the interaction of PKA-RI with cTnT nearby the substrate cTnI located at the thin filament or with myomegalin, which has been proposed as a direct interaction partner of cMyBP-C. Both proteins were reported as AKAPs with the potential to anchor type I PKA at the cross-bridge bearing region of the sarcomere [56,57]. Other AKAPs that have been reported to exist close to the myofilament compartment, such as myospryn and synemin, appear to specifically interact with PKA-RII, excluding an effect of interdisulfide formation on binding affinity due to the absence of the responsible cysteines in the amino acid sequence [58,59]. Oxidation-mediated alteration of the affinity of PKA for its AKAPs that results in the subcellular translocation of the holoenzyme and subsequent release of the active catalytic subunit into substrate vicinity represents a possible order of events. An interesting question that to our knowledge has not been answered yet is, whether AKAP oxidation itself poses a prerequisite for the inter-action with PKA-RI. As they represent important scaffold regulators of PKA activity within the cell, the oxidation of an AKAP could potentially be involved in the observed translocation effect and thus mediate the cellular response to oxidative stress (Figure 3).

## 5. Oxidative Post-Translational Modification of PKA-C

Another layer of complexity to the redox regulation of PKA is added by the fact that also the catalytic subunit of PKA is susceptible to oxidative modification. Only two cysteine residues exist within PKA-C, Cys199 and Cys343 (human Cα sequence), and both are susceptible to oxidation [62,63,64] (Figure 3A). Cys199 is the more reactive residue, which is reflected by its lower *p*Ka value. It is located within the activation loop near the active site of the kinase, where it interacts with the hydrophobic residue of the substrate sequence that is present at position + 1 of the PKA consensus sequence [65]. Although Cys199 is not directly involved in the catalytic activity of PKA-C, the modification of the residue by thiol-modifying agents or oxidants results in inhibition of kinase activity [63,64,66,67,68]. In response to diamide-exposure, two different oxidative post-translational modifications, namely S-glutathionylation at Cys199 and intradisulfide bond formation between Cys199 and Cys343, have been observed for PKA-C [66]. Experiments from our group with recombinant bovine PKA-C showed that the HNO donor NCA supported these observations. Here, inhibition of catalytic activity was accompanied by a PKA-C band migrating at a lower molecular weight in native Western immunoblots, likely representing a Cys199-Cys343 intradisulfide-containing form of the enzyme [61]. Intradisulfide formation is only possible by structural rearrangement of the C-tail. This reorganization influences the kinase structure as shown before [44,66]. Inhibition of kinase activity by Cys199-Cys343 intradislfide formation might be explained by the C-tail blocking the catalytic cleft as a result of rearrangement. In a cellular context, however, this oxidative modification has not been observed yet, thus its physiological relevance remains disputable and suggesting that other modifications such as S-glutathionylation of Cys199 prevail in vivo [66]. Another possibility is that the applied techniques were not suitable to detect this modification in a complex cellular context. Recently, an interesting study postulated reversible inhibitory oxidation of a conserved cysteine in the +2 position adjacent to a critical threonine phosphorylation site in the “T-loop” of kinases of the AGC family and others as a universal regulatory mechanism governing physiological and pathological cellular responses to oxidants [69]. The authors expressed a redox-dead Cys199Ser mutant of the catalytic subunit of PKA in cells and analyzed substrate phosphorylation using a pan-PKA phospho-substrate antibody. Convincingly, they showed a discrepant phosphorylation pattern in mutant versus wildtype transfected cells. Future experiments should, however, embrace simultaneous oxidation of PKA-RI into the interpretation of the results.

In this context, it may seem contradictory that the oxidation of PKA-RI is ascribed to kinase activation, although the same modification exerts an inhibitory effect on the catalytic subunit of PKA. The spatiotemporal order of events might play a role in this scenario. Holoenzyme assembly protects PKA-C Cys199 from oxidative modification [63]. Oxidation-mediated translocation has been observed for regulatory [43] and also catalytic PKA subunits, suggesting that PKA translocates as a holoenzyme [44]. If the hypothesis holds true that PKA is guided towards sarcomeric substrates by the increased interaction of dimerized PKA-RI with an AKAP, Cys199 does not become available for oxidation until PKA-C subunits are released in substrate vicinity. In line with previous reports [62], our in vitro data showed that preincubation with ATP protected PKA-C from oxidative kinase inhibition and reduced the occurrence of intradisulfide formation detected by the mass shift in native Western immunoblots, most likely due to conformational restriction. As cross-bridge cycling represents the process that consumes the most ATP in cardiac muscle [70], it is likely that oxidants have to compete with ATP for access to the activation loop of free PKA-C, where Cys199 is located (Figure 3A). Consequently, the inhibitory effect of oxidation on PKA-C in this scenario is considered subordinate to activating PKA-RI disulfide formation, resulting in the observed net outcome of protein phosphorylation due to increased PKA activity [43,44]. Oxidation-induced translocation of PKA to other subcellular microdomains, where high concentrations of ATP cannot protect PKA-C from oxidation, may result in a more profound inhibitory effect on PKA and should be addressed in future research efforts. Interestingly, investigations on type II PKA unveiled that modification of Cys199 did not disrupt the holoenzyme complex of PKA-C with the regulatory subunits [64]. Based on this finding, it can be speculated whether PKA-C, once oxidized, can be locked in this inactive state when catalytic subunits re-assemble with PKA-R before the modification at Cys199 is reduced. By denying access to components of the cellular antioxidant defense machinery, this mechanism could, on short notice, diminish subsequent kinase activation via PKA-RI oxidation. To date, however, this theory lacks experimental evidence and should be considered at this timepoint as purely hypothetical.

Although PKA is considered one of the best characterized protein kinases in the heart, plenty aspects remain unknown. This involves the impact of oxidative post-translational modifications on holoenzyme conformation, kinase activation, activity and cellular functions. The oxidant-mediated impact on PKA at a given time and cellular location is the end-product of orchestrated events based on the susceptibility of both, regulatory and catalytic PKA subunits for thiol oxidation. In cooperation, these modifications have a significant influence on the phosphorylation of critical cardiac myocyte proteins and thus cardiac contractility, but also other cellular PKA functions. Thorough research will be required in the coming years to gain a better understanding of the (patho)physiological PKA redox regulation, its interplay with the classical cAMP-dependent activation pathway, the consequences for cardiac performance and how this knowledge can be applied to develop novel therapies for cardiac diseases.

## 6. Regulation of PKA Signaling by Protein Phosphatase 2A

In humans, the 518 genes of the kinome that were identified to putatively encode for protein kinases are opposed by only 189 genes encoding for protein phosphatases [71,72]. While the largest group of protein kinases is specific for serine/threonine, only a small number of protein phosphatases exists to counteract their activity. This is because the majority of protein phosphatases are specialized to dephosphorylate tyrosine residues [73]. The numerical inferiority of serine/threonine phosphatases compared to the corresponding protein kinases is balanced by the combination of a conserved core enzyme with a variety of regulatory subunits, resulting in a high diversity of phosphatase holoenzymes.

PP2A is the major serine/threonine phosphatase in eukaryotic cells and highly abundant in the heart [74]. It is involved in the dephosphorylation of several cardiac myocyte PKA substrate proteins that participate in excitation-contraction coupling, such as the L-type Ca^2+^ channel [75,76], cMyBP-C [77], cTnI [78], most likely the RyR2 [25,79,80,81] and to a lesser extent PLN [82]. Due to its substrate spectrum, PP2A activity has a decisive influence on cardiac myocyte Ca^2+^-cycling and cardiac contractility. Moreover, PP2A is an important regulator of mitogen-activated protein kinase (MAPK) signaling and thus plays a critical role in cardiac remodeling, development, physiological function and the manifestation of cardiac pathologies [83,84,85,86,87,88,89].

The heterodimeric PP2A core enzyme consists of a catalytic (PP2A-C) and a scaffold subunit (PP2A-A), which both exist as an α or β isoform [90,91]. PP2A-C and PP2A-A α and β isoforms are both ubiquitously expressed and share ~97% and ~87% sequence identity, respectively [90,91,92]. Despite the high similarity in sequence, PP2A-C isoforms are not redundant. Observations that PP2A-Cα mRNA is more abundant than mRNA encoding for the β isoform in porcine tissues including the heart [74,93], and the fact that a knockout of PP2A-Cα in a mouse model is embryonically lethal [94], suggest essential isoform-specific functions for PP2A-Cα and β within the cell.

Combination of the core enzyme with one of at least 18 regulatory B subunits (PP2A-B) forms the fully active PP2A holoenzyme (Figure 3B). The regulatory subunits can be divided into four groups B/B55/PR55, B’/B56/PR56/PR61, B’’/PR48/PR72/PR130 and B’’’/PR93/PR110 and display a surprisingly low similarity in sequence [95]. Within the heterotrimeric enzyme, the PP2A-B subunits mediate subcellular localization and substrate specificity of the holoenzyme and represent the major regulatory mechanism that determines PP2A spatiotemporal activity in the cell [96,97]. For example, the recombinant isotypes γ and the δ of the B56 regulatory subunit were found in the nucleus, whereas B56α, β and ε were located within the cytoplasm in experiments involving CV-1 monkey kidney cells suggesting distinct roles in subcellular phosphatase targeting [98]. In cardiac myocytes, PP2A targeting by B56α was shown to depend on the interaction of B56α with ankyrin-B that is required for proper subcellular placement and orientation of ion channels [99]. Consequently, B56α is assumed to guide PP2A activity towards the membrane bound Na^+^/K^+^-ATPase and Na^+^/Ca^2+^-exchanger [97,99,100]. In adenovirally transduced neonatal rat ventricular myocytes, recombinant B56α displayed a striated cytosolic pattern typical for sarcomeric localization [101]. In line with this observation, B56α was detected in the myofilament-containing subcellular fraction of adult rat ventricular myocytes [102]. Notably, myofilament B56α levels decreased upon treatment with the β-AR agonist isoprenaline, suggesting B56α to regulate PP2A activity at myofilament substrate proteins in dependence of neurohumoral stimulation.

## 7. Regulation of PP2A by Post-Translational Modification

In addition to holoenzyme composition itself, numerous post-translational modifications (PTM) have been identified that additionally impact on subunit assembly and PP2A-mediated substrate dephosphorylation. Several members of the B’/B56 group of regulatory PP2A subunits have been identified as phosphoproteins [98,103,104]. While B’/B56 subunit phosphorylation has been associated with holoenzyme dissociation and inactivation [86], several studies have demonstrated that the phosphorylation of B56δ at Ser37, Ser566 or Ser573 by PKA and other kinases enhances phosphatase activity [105,106,107], supposedly by altering the interaction with the core enzyme [104].

Also, the PP2A catalytic subunit is prone to PTM. Nitration, methylation and phosphorylation of PP2A-C occur within its conserved C-terminus and affect PP2A activity by altering holoenzyme assembly. Various studies described PP2A-C to be susceptible to NO-mediated nitration at Tyr284, which is assumed to elevate PP2A activity by inducing the dissociation of PP2A-C from its respective scaffolding subunit [108,109,110,111]. PP2A methylation at the C-terminal residue Leu309 by the PP2A-specific leucine carboxyl methyltransferase (LCMT1 or PMT) has been generally associated with enhanced phosphatase activity [112,113]. The modification is reversed by the methylesterase PME-1 [114], thereby modulating the affinity for certain PP2A-B subunits [115,116]. However, there is contradiction regarding the class of regulatory subunit that depend on PP2A-C methylation [117,118,119]. Similarly, phosphorylation of PP2A-C at Tyr307 and potentially Thr304 is associated with the inhibition of the phosphatase by impeding the interaction with multiple B’/B56 or B/B55 subunits, respectively [84,119,120,121]. Notably, although PP2A is a Ser/Thr phosphatase, it is able to restore its own catalytic activity by PP2A-C auto-dephosphorylation of Tyr307 [122,123]. In summary, the data of these studies highlight that specific PTM-related events are required for PP2A-B subunit interaction with the PP2A-C C-terminus to allow assembly with the core enzyme, generating a holoenzyme with specific features.

Another PTM that has not yet received much attention in the regulation of PP2A activity is the reversible oxidation of PP2A-C cysteine thiols. A number of studies showed that PP2A-C activity was inhibited by treatment with various thiol-modifying agents including maleimide and mercurial compounds [124,125]. Additionally, it was reported that oxidants including H_2_O_2_ and oxidized glutathione exerted an inhibitory effect on PP2A, while the activity was restored by exposure to reducing agents [85,126,127,128,129,130,131]. Investigations on the molecular mechanisms of the PP2A inhibitors okadaic acid, phoslactomycin, fostriecin and microcystins had consistently identified Cys269 of PP2A-C as the binding site, suggesting that modification at this site likely interferes with PP2A catalytic activity [132,133,134,135]. Accordingly, oxidative intradisulfide formation between the residues Cys266 and Cys269, which localize in a redox sensitive CXXC motif, was suggested to act as a redox switch that mediates reversible oxidation-dependent PP2A inhibition [131,136]. In line with this hypothesis, a faster migrating band that was abolished by the addition of DTT representing intradisulfide-containing PP2A was detected by native Western immunoblotting in rat brain homogenates [131]. Another pair of vicinal thiols, namely Cys50 and Cys55, exists within PP2A-C. However, modification of these residues was not detected in a study aiming to label and identify reactive thiols by an unbiased mass spectrometry approach [125].

In rat brain cerebral cortex, Foley et al., further highlighted the high reactivity of PP2A-C thiols by demonstrating that spontaneous oxidation without the addition of an oxidizing agent was sufficient to induce disulfide bond formation [131,136]. Furthermore, even under stringent experimental conditions designed to prevent protein oxidation du-ring sample preparation, a small fraction of PP2A-C was found to contain DTT-reversible disulfide bonds, indicating such modifications likely to occur in vivo [136]. This is supported by studies showing that in neuronal as well as in tumor cell lines, the intracellular generation of ROS and subsequent ROS-mediated inhibition of PP2A induces apoptosis via activation of the MAPK pathway [88,137]. Another argument supporting PP2A-C disulfide-mediated inhibition to represent a crucial cellular regulatory mechanism was provided by the observation that PP2A from rabbit skeletal muscle was associated with nucleoredoxin, potentially via an interaction with the PP2A catalytic subunit [138]. As a member of the thioredoxin family, the oxidoreductase nucleoredoxin is involved in redox regulation and may thus impact on the oxidation-mediated function of PP2A-C [139].

Only little is known about the physiological functions of PP2A-C oxidation in cardiac myocytes. In our recently published study on the effects of oxidants on the phosphorylation of cardiac myocyte proteins, we observed that HNO donor compounds induced protein phosphorylation via oxidation-dependent translocation of PKA towards critical myo-filament substrates [44]. Further research revealed that upon HNO-treatment, not only PKA, but also PP2A-C was accumulating within the myofilament-containing fraction of cardiac myocytes. In line with previous reports, the assessment of PP2A activity from adult rat ventricular cardiac myocyte preparations showed an inhibitory effect following oxidation, while its activity was restored upon addition of the reducing agent DTT.

As outlined before, phosphorylation and methylation of the PP2A-C C-terminus alter PP2A activity via modulation of the interaction with PP2A regulatory subunits. Accor-ding to these observations, it has been suggested that PP2A-C oxidation may exert its inhibitory effect via an influence on the well-established PTM of the C-terminus [140]. Al-though an impact of PP2A-C intradisulfide formation on the interaction with certain re-gulatory subunits cannot be excluded, data supporting this hypothesis is scarce. Various studies reported enhanced PP2A-C phosphorylation coinciding with reduced methylation in response to elevated ROS in different cell types, conditions that are expected to reduce PP2A activity [88,137,141]. However, this is likely to only represent a correlation, rather than a causal relationship. An oxidant-induced increase in PP2A-C phosphorylation could, for example, be explained by the oxidation-mediated inhibition of the previously reported ability of PP2A to auto-dephosphorylate [122,123]. In addition, the exposure to ROS is likely to influence a multitude of signaling pathways in cells that may lead to alterations in PP2A PTM as well. DeGrande et al. showed that altered levels of PP2A-C phosphorylation and methylation were detected in H_2_O_2_-treated murine ventricular cardiac myocytes, but no differences were observed in cardiac fibroblasts [141]. This suggests cell type specific responses to ROS rather than PP2A-C oxidation itself to be responsible for changes in these PP2A-C PTM levels, although these modifications may as well contribute to the ROS-induced inhibition of PP2A. Experiments performed by our group on human recombinant PP2A-C reported that the exposure to HNO significantly reduced PP2A-C-mediated dephosphorylation of the fluorogenic substrate DiFMUP, corrobora-ting the assumption that oxidation at Cys269 indeed had a direct negative effect on the catalytic activity of PP2A-C subunit in vitro [44]. This presumption was further supported by the fact that Cys269, which is also the target of several PP2A inhibitors [132,133,134,135], is located in vicinity to the PP2A-C active site [117].

Interestingly, the susceptibility of PP2A to oxidative inhibition varies between cell types, which is probably due to differences in their antioxidant capacity. While H_2_O_2_ reportedly induced PP2A oxidation in brain [94], Caco-2 cells [127] and fibroblasts [129], H_2_O_2_ may not be able to oxidatively inhibit PP2A-C in cardiac myocytes. Pharmacological treatment of adult rat ventricular myocytes by our group showed, in contrast to HNO donors and diamide, no H_2_O_2_-mediated (100 µM) alterations in the phosphorylation status of the PP2A substrate proteins cMyBP-C, cTnI or PLN [44]. Two publications from the Hofmann group also investigated PP2A activity in rat cardiac myocytes upon exposure to H_2_O_2_. They showed that overall PP2A activity remained unchanged in cells treated with H_2_O_2_. They even reported increased activity for a PP2A moiety that was precipitated in association with its substrate ERK [142] or PP2A that was collected with the myofilament-containing fraction [143], respectively. This elevation in PP2A activity was attributed to non-oxidative PTM, especially enhanced PP2A-C methylation [143]. In contrast to these findings, DeGrande et al. reported a decrease in methylation of PP2A-C in murine cardiac myocytes treated with 75 µM H_2_O_2_ [141], highlighting that further research is required to better understand PP2A PTM regulation by ROS. Importantly, the data also denote the inability of H_2_O_2_ to reproducibly induce oxidative PP2A-C inhibition in cardiac myocytes at the concentrations applied in these studies. A potential explanation could be that the inhibitory effect was masked by other H_2_O_2_-induced cellular events.

In our experiments, oxidation-dependent translocation of PP2A into the myofilament-containing fraction of cardiac myocytes occurred [44]. It, however, remains uncertain to date whether this change in subcellular localization was induced by PP2A-C intradisulfide bond formation altering the affinity for certain regulatory subunits. Importantly, PEG-switch experiments provided convincing evidence that the B56α regulatory subunit that was accumulating within the myofilament fraction alongside with PP2A-C was subject to DTT-reversible oxidative modification. B56α had been detected within the myo-filament-containing fraction of cardiac myocytes before and is assumed to guide PP2A towards its sarcomeric substrates [102]. However, the physiological significance of B56α oxidation for PP2A-targeting remains unknown. It is of note that, to our knowledge, the susceptibility to oxidative modification of the B56α regulatory subunit has not been observed before. Oxidative modification of another member of the B’/B56 family, B56δ, was previously described by Low et al. [144]. Here, peroxynitrite-mediated nitration of substrate-associated B56δ at Tyr289 prevented the assembly with the PP2A core enzyme. Thus, oxidative modification of B’/B56 subunits may represent a regulatory mechanism for PP2A in cardiac myocytes and other cell types. Although PP2A-C was shown to be highly susceptible to oxidization under certain intracellular conditions [131,136], PP2A-C may remain active to be guided towards specific substrates by oxidized B56α or other oxidation-susceptible B’/B56 regulatory subunits that remain to be identified. It is also important to mention that exclusively members of the B’/B56 group of regulatory PP2A subunits have been identified as phosphoproteins in vivo [98,104]. In essence, these findings point towards B’/B56 regulatory subunits to be highly regulated and versatile PP2A-Bs that, by phosphorylation and oxidation, allow efficient adaptation of PP2A activity to the dynamical changes in intracellular conditions.

To unravel the actual role of PP2A-C and PP2A-B oxidation on PP2A subunit affinity, holoenzyme localization and phosphatase activity as well as the underlying regulatory mechanisms, more detailed research employing animal and cell models expressing redox-deficient phosphatase subunits are needed. Thus far, the available data suggest that PP2A subunit oxidation represents a previously underrated extension in the spectrum of PTM that contributes to the fine-tuned spatiotemporal regulation of PP2A activity in the heart and, most likely, many other tissues.

## 8. Regulation of PKA Signaling by Protein Phosphatase 1

Protein phosphatase 1 (PP1) is a highly conserved Ser/Thr-phosphatase, whose action is essential for unperturbed cardiac function [145,146,147]. Like PP2A, PP1 mediates the restoration of protein phosphorylation to basal levels, normalizing cardiac contractility after neurohumoral stimulation and PKA activation. PP1 shares several cardiac myocyte substrates with PP2A. PP1 dephosphorylates cMyBP-C [77,148,149], RyR2 [25,150], cTnI and cTnT of the troponin complex [78,151] and is considered to be the main phosphatase to regulate the phosphorylation status of PLN [82,152,153]. Nonetheless, there is no redundancy of the two phosphatases. This is highlighted, by the observation that approximately 70% of phosphatase activity dephosphorylating PLN was attributed to PP1 [82]. More-over, a study that applied phosphopeptide mapping showed that dephosphorylation of cTnT and cTnI by PP2A occurred in a uniform manner, but for PP1 was characterized by differences in phosphatase activity towards certain phosphorylated residues [78]. Site-specificity within substrate proteins as evidenced for cTnT and cTnI demonstrated the distinct functions of PP2A and PP1 in the heart [97].

The PP1 holoenzyme consists of a catalytic subunit that assembles with a multitude of regulatory proteins, which determine phosphatase substrate specificity, regulate subcellular targeting and were also proposed to impact on protein stability [153,154,155]. In mammals, the catalytic subunit exists in six isoforms that possess an amino acid identity of approx. 90% and are encoded by three genes: PP1α with its splice variants α_1-3_, PP1β (also called δ) and the PP1γ splice variants γ_1_ and γ_2_ [156,157,158]. While PP1γ_2_ is predominantly expressed in testis [159], the other isoforms are ubiquitously expressed. The differ-rences between PP1 isoforms has been described (for a detailed review on PP1 isoforms see [160]). PP1 can be phosphorylated at Thr320 by cyclin-dependent kinases, which inhibits phosphatase activity [121,161]. This modification, however, seems to be meaningful mainly for cell cycle regulation, especially during mitosis [162]. As a common feature of all protein phosphatases of the Ser/Thr family, two metal ions exist within the active site of PP1, which are required as a cofactor for catalytic activity. These cations, usually Mn^2+^, are coordinated by six amino acids (Asp64, His66, Asp92, Asn124, His173 and His248), which are highly conserved among eukaryotic protein phosphatases [163].

An estimated amount of over 100 different proteins can interact with the PP1 catalytic subunit to assemble the PP1 holoenzymes. Although a regulatory role for many of these interacting proteins has been discovered, for some it remains unclear, whether they possess a regulatory function, represent PP1 substrates or both [153,155]. The interaction of most regulatory proteins with PP1 occurs via a conserved RVxF motif that interacts with a hydrophobic groove on the PP1 surface [164,165].

Two prominent examples for PP1 regulatory proteins are inhibitor-1 (I-1) and inhi-bitor-2 (I-2) which, according to their name, inhibit PP1 activity, but with opposing dependence on phosphorylation. While I-1 requires phosphorylation to exert its inhibitory function, phosphorylation of I-2 temporarily abolishes its inhibitory effect on PP1 [153]. Mouse models overexpressing either of the two inhibitor proteins were generated to demonstrate the physiological importance of PP1 for cardiac function [146,147]. In both studies, the active inhibitors reduced overall PP1 activity, resulting in elevated PLN phosphorylation and augmented cardiac contractility. Various publications showed that in cardiac myocytes, I-1 activity is tightly coupled to β-AR signaling. I-1 is activated by PKA-mediated phosphorylation at Thr35 following β-AR stimulation by isoprenaline and in this way contributes to enhanced cardiac contractility in response to neurohumoral activation [166,167,168,169,170,171]. Thr35 dephosphorylation catalyzed by PP2A and PP2B (calcineurin) represents a potential link between Ca^2+^- and cAMP-dependent signaling [172]. Additional phosphorylation sites within I-1 were reported to exist at Ser67 and Thr75. Despite contradicting observations concerning the effect of pSer67 on PP1 function [173,174], it is now assumed that phosphorylation at both, Ser67 and Thr75 by PKCα, enhances PP1 activity by impeding Thr35 phosphorylation [175,176,177,178].

## 9. Regulation of PP1 by Post-Translational Modification

In addition to the myriad of regulatory proteins that regulate PP1 function in the cell, PP1 activity is also modulated by oxidative modification. In 2002, Sommer et al. [128] investigated the susceptibility of different Ser/Thr phosphatases to oxidative inhibition. The authors demonstrated an inhibitory effect of H_2_O_2_ on PP1 from SK-N-SH cell lysates, indicating that PP1 may be susceptible to direct oxidative modification [128]. An important observation was that ascorbic acid, a biological reductant that can interact with metal ions, restored PP1 activity, when applied in combination with the thiol reducing agent DTT after H_2_O_2_-mediated phosphatase inhibition. This finding gave rise to the hypothesis that the inhibitory effect of H_2_O_2_ on PP1 may be caused by a combination of cysteine modifications and the oxidation of metal ions located within the catalytic center of PP1. A study published shortly after reported that H_2_O_2_-treatment of differentiated PC12 cells reduced PP1 activity and simultaneously resulted in enhanced phosphorylation levels of the PP1 substrate eIF2α [179]. This effect could be reversed by the antioxidant compounds N-acetylcysteine (NAC) and reduced glutathione (GSH), and reintroduced by the addition of the PP1 inhibitor tautomycin, providing further evidence that oxidation could interfere with PP1 activity. Notably, the inhibition of PP1 could not be abrogated by the thiol reducing agents β-mercaptoethanol or DTT. Under the assumption that NAC and GSH may reduce both, oxidized thiols and redox-active metal ions, this result argued for the oxidation of metal ion cofactors as the major mechanism underlying oxidative PP1 inhibition. This speculation was further supported by another publication, which showed that PP1-mediated dephosphorylation of eIF2α was reduced by ROS generated by Nox4 in H9c2 cardiomyoblasts [180]. The data demonstrated the formation of an endoplasmic reticulum-associated complex comprising Nox4, PP1 and the PP1 regulatory subunit GADD34, with the local release of H_2_O_2_ by Nox4 leading to in vivo inhibition of PP1. Neither DTT nor GSH could restore PP1 activity in this study, while ascorbic acid dose-dependently reversed PP1 inhibition. Biophysical experiments further supported the hypothesis that the oxidation of catalytic Mn and/or Fe ions may be the underlying mechanism for PP1 inactivation. Finally, the replacement of amino acid residues Asp64 or Asn124, which participate in the coordination of the cofactor metal ions, for Asn or Asp, respectively, increased the H_2_O_2_-induced inhibitory effect. Importantly, the inhibition of PP1 activity by Nox4 was also shown to play a role in the heart, as this process was identified to limit infarct size in murine hearts after ischemia/reperfusion. Another observation that may be interpreted in support of thiol modification-independent inhibition of PP1 is the absence of a cysteine residue corresponding to Cys269 in PP2A, which mediates PP2A inactivation by several inhibitory agents [132,133,134,135]. Okadaic acid (OA), which is a potent inhibitor of both, PP2A and PP1, binds PP1 via a hydrophobic groove near the active site, also invol-ving additional residues such as Phe276 [181]. Interestingly, the replacement of Phe276 in PP1 for Cys attenuated the inhibitory constant (*K_i_*) of OA and is likely to partially explain the higher affinity of OA for PP2A [181,182]. Thus, PP1 may lack a redox-sensitive cysteine residue that, in the closely related phosphatase PP2A, represents the main site for oxidative inhibition.

Despite the fact that experimental evidence exists that points towards the metal ions of PP1 in the catalytic center as the site of inhibitory oxidation, there is also data that convincingly supports the existence of oxidative cysteine disulfide bond formation. PP1 isolated from rabbit skeletal muscle could be inactivated by alkylating, oxidizing and mercaptide-forming thiol-reactive reagents [124]. Analysis of inhibition kinetics suggested that the modification of a single residue was responsible for the inhibitory effect. Inhibition could be abolished by DTT and β-mercaptoethanol and to some extent by GSH and pointed towards reversible oxidative inhibition of PP1 via thiol modification. ROS-genera-tion in human diploid fibroblasts during senescence was found to be responsible for the inhibition or Ser/Thr phosphatases and elevated ERK1/2 phosphorylation [129]. Phosphatase inhibition by H_2_O_2_ in juvenile cells was reversible by DTT, β-mercaptoethanol and NAC, arguing for the involvement of thiol oxidation. However, a lack in discrimination between PP1 and PP2A activity in these experiments did not allow to definitely assign the effect to one or the other phosphatase. Using recombinant PP1α, a combination of DTT and ascorbate resulted in profound restoration of activity after H_2_O_2_-mediated inactivation, suggesting that metal ion oxidation, but also the modification of cysteine thiols was responsible. PP1α contains 13 cysteine residues and only slight deviations exist between isoforms. By mass spectrometry analysis of recombinant PP1α, Cys62 and Cys105 were detected in the oxidized sulfonic acid (-SO_3_H) state. Although this supported the assumption of H_2_O_2_-mediated PP1 thiol oxidation, it is a surprising result as sulfonic acid modification is considered to be irreversible and indicates over-oxidation, damage and dysfunction.

In brain, oxidative stress had been associated with neuronal damage and schizophrenia in earlier studies [183,184]. An investigation of rat brain extracts eventually identified PP1 amongst the proteins that contained disulfide bonds, which were reducible by the mild reducing agent tris(2-carboxyethyl)-phosphine (TCEP) [185]. A mass spectrometry approach that used a mechanism of data evaluation especially designed for the identification of disulfide bonds, detected interactions between Cys39-Cys127 as well as Cys127-Cys127 of recombinant PP1α with increasing concentrations of H_2_O_2_ [186]. Both types of bonds suggested the formation of PP1 interdisulfide dimerization, since the distance between Cys39 and Cys127 was assumed too far to form an intradisulfide bond. In accordance with the formation of PP1 dimers, Western immunoblot analysis in neonatal rat cardiac myocytes revealed an extra band migrating at about twice the molecular weight of PP1, which grew in intensity upon treatment with diamide. Notably, by both, mass spectrometry and immunoblot analysis, disulfide formation was already present under basal conditions, pointing towards a high sensitivity of the cysteine residues involved. The extensive study by Singh et al. further suggested that S-glutathionylation may promote the formation of different transient intradisulfide bonds within PP1 [186]. In this context, Cys39 was thought to be of particular importance as it may protect other cysteine residues from oxidation. The formation of intradisulfides, however, was largely dependent on the activity of glutathione S-transferase (GST) and mainly observed when GST-tagged PP1 was applied in the presence of GSH. Furthermore, the authors speculated that His248, which is considered as one residue that cooperates the active center metal ions [163], may play a minor role, but, by being oxidized itself, could have a protective or regulatory role for cofactor metal ion oxidation.

It is currently not possible to definitively conclude, which oxidative modifications of PP1 occur under physiological conditions in cardiac myocytes, but the importance of disulfide formation is doubtful. Cys127 was the main site involved in interdisulfide bonds reported by Singh et al. [186]. However, Santos and colleagues did not detect any differen-ces of PP1 activity or H_2_O_2_-induced inactivation when Cys127 and Cys273 were exchanged to Ser in recombinant PP1 [180]. A protective role of interdisulfide bonds concerning the oxidation of catalytic metal cofactors therefore seems unlikely. But while disulfide dimer formation of PP1 may not directly impact on the catalytic activity of PP1, it cannot be ruled out that it may have an influence on the interaction with certain PP1 re-gulatory proteins and may shield the active surface in the absence of a substrate, as it had been hypothesized by Singh and colleagues. Transient disulfide bonds involving Cys39 and Cys127 were further speculated to protect PP1 from irreversible inhibition by over-oxidation and may exist in vivo. In fact, many publications show that PP1 activity could be restored even after exposure to high concentrations of oxidants [128,179,180]. However, it remains to be elucidated in future experiments, whether this ability to restore PP1 activity is the result of a protective impact mediated by disulfide formation. Moreover, the observation from Singh et al. that fewer disulfide bonds were detected when Mn^2+^ ions were present in the mass spectrometry experiments argues for the active center metal ions as the major target for oxidation [186]. It is also of note that Cys62 and Cys105, which had been identified as particularly susceptible to oxidation by Kim et al. [129] have not been detected as prominent targets for oxidation by Singh et al. [186]. In summary, convincing data suggest the oxidation of metal ions that act as cofactors during PP1 catalytic activity to be the most important regulatory targets for PP1 oxidation. The detection of numerous kinds of disulfide bonds, however, indicate that many cysteine residues of PP1 are susceptible to thiol oxidation. Which of these bonds actually form in cellulo and how they affect PP1 activity in vivo should be investigated in future research to gain a better understanding of how PP1 function is regulated by oxidants.

Substantial evidence for PP1 oxidation as a physiological regulatory process in the heart is provided by the fact that it can be induced by in vivo-generated ROS. An inhibitory effect on Ser/Thr phosphatases via ROS was determined in senescent human diploid fibroblasts [129]. Other publications presented related results based on Nox2 and Nox4, which are the main types of NADPH oxidases in cardiac myocytes [187]. As discussed above, ROS release by Nox4 during H_2_O_2_-induced oxidative stress was shown to inhibit PP1 [180]. Interestingly, a similar mechanism was reported by the same group for Nox2. Transgenic mice with a cardiac myocyte targeted overexpression of Nox2 displayed enhanced contractile function with accelerated contraction and relaxation upon administration of Nox2-activating angiotensin II generation, when compared to wild type animals [188]. This effect was found to rely on increased phosphorylation levels of PLN as a consequence of Nox2-dependent oxidative inhibition of PP1, leading to increased Ca^2+^-cycling at the SR. Interestingly, although PLN Ser16 phosphorylation after AngII-exposure was shown to rely on PKA, no other phosphorylation site of PP1/PKA substrate proteins displayed alterations. It was therefore speculated that only a small PLN-associated subpopu-lation of PP1 was susceptible to inhibition via Nox2. This matched with the release of ROS by Nox4, which was shown to occur locally at a microdomain level [180]. The two studies investigating Nox-induced impact on PP1 activity thus demonstrated redox-regulation of PP1 in living cells and highlighted the importance of PP1 oxidation as an important regulatory mechanism for cardiac function.

An indication that PP1 oxidation may also mediate subcellular localization is provided by experiments showing DTT-reversible PP1 accumulation in the myofilament fraction of cardiac myocytes after exposure to the HNO donor NCA [44]. Whether this translocation happens due to oxidation-induced alterations concerning the interaction with regulatory subunits or potentially involves the oxidation of regulatory proteins remains unknown to date. No impact of PP1 oxidation on holoenzyme composition had been observed after H_2_O_2_-exposure in a previous study [179].

During β-AR stimulation, PKA signaling is tightly coupled to the regulation of its opposing phosphatases. Phosphorylation of the PP1 inhibitor I-1 or the B56δ regulatory subunit of PP2A by PKA represents regulatory feedback mechanisms that can further modulate the phosphorylation of PKA/PP1/PP2A substrate proteins such as PLN or cMyBP-C in cardiac myocytes [107,171,189]. In line with the findings discussed before that PKA can be activated by oxidation, Singh et al., showed enhanced PKA-mediated phosphorylation of the PP1 inhibitor I-1 upon treatment with H_2_O_2_ in neonatal rat cardiac myocytes [186]. In adult rat cardiac myocytes, our recently published results presented an interplay of oxidation-dependent changes in PKA and PP2A activity after exposure to HNO. These results highlight that the susceptibility of PKA, PP1 and PP2A to oxidative modification adds another level of complexity to the crosstalk between these three major regulators of cardiac contractility. To date, several publications reported that treatment of cardiac myocytes with oxidants caused alterations in the phosphorylation status of substrate proteins including PLN and cMyBP-C that are shared by PKA/PP2A/PP1 [43,44,186,188]. However, in many cases it remains uncertain whether the resulting phosphorylation levels are a result of altered kinase or phosphatase activity, or an interplay of both. The knowledge that is currently available emphasizes that tremendous complexity exists concerning the events that orchestrate the net phosphorylation of functionally criti-cal phosphoproteins in cardiac myocytes, with protein oxidation representing an additional adjustment screw that has so far been little explored. With the aim to open up new possibilities for the development of therapeutic strategies to face cardiac disease, this network of redox-regulated enzymes needs to be fully unraveled.

## 10. Therapeutic Modulation of Kinase/Phosphatase Signaling

The overarching question remains whether pro-oxidative modulation of the PKA signaling cascade might be exploited as a druggable target with therapeutic implications. Since increased ROS production is associated with adverse development of cardiovascular disease, administration of antioxidants was presumed as a therapeutic strategy. Des-pite promising observational studies and preclinical results [190], clinical trials to test if dietary antioxidants, such as vitamin E and vitamin C, have a beneficial effect in the treatment of cardiovascular diseases and might improve the patients’ disease condition, failed to provide a beneficial cardiovascular outcome [191,192,193,194,195,196]. In the case of vitamin E, long-term administration was even associated with increased risk of heart failure development [197]. Therefore, antioxidant dietary therapy for cardiovascular disease has not yet lived up to the scientific expectations and other avenues to manipulate ROS production in the heart should be carefully evaluated.

Interestingly, compounds that induce protein oxidative modifications have been proposed as a potential therapy for the treatment of cardiovascular diseases. A prominent example is HNO. The HNO donor BMS-986231 (alternatively known as cimlanod or CXL-1427), a pro-drug of CXL-1020, is currently tested in clinical trials for the treatment of patients with acute decompensated heart failure [198,199,200]. The beneficial mechanism of its cardiovascular actions combines vasorelaxation [201,202,203] and enhancement of cardiac inotropy. The latter has been attributed to general disulfide induction in oxidant-susceptible proteins that culminate in the enhancement of sarcomere shortening and cardiac myocyte relaxation. This includes an increase in Ca^2+^-reuptake into the SR and an acceleration of cross-bridge cycling by disulfide crosslinking of proteins localized to the SR—PLN and SERCA2a—and the myofilament—tropomyosin and the myosin light chains—compartment [204,205,206,207,208]. Recently, disulfide formation in PKA and PP2A subunits has been described to contribute to this scenario and to occur independently of β-adrenoceptor activation [44]. However, the multitude of proteins that are modified by HNO donors reflects the lack of selective targeting exerted by these pro-oxidant compounds that require their clinical application to patients with caution.

An alternative, but undoubtedly promising strategy for the inspiration of novel therapeutics is the development of selective compounds that may target exclusive sites within the protein kinase “cysteinome” [209,210]. The aim is the rational design of selective modulators that adduct covalently to the highly electrophilic thiol group of cysteines in and around the ATPbinding site. This way kinase or phosphatase enzymatic activity would be therapeutically regulated. That this strategy is not impossible has been demonstrated by a “clickable” inhibitor of the p90 ribosomal S6 kinase (RSK) isoforms 1 and 2, FMK, a fluoromethylketone compound, which was designed to bind irreversibly to Cys432 (numbering refers to RSK1). FMK-binding exerts a sterical hindrance on the “gatekeeper” Thr489 residue in RSK, consequently preventing access of ATP to its C-terminal kinase domain [211]. Deregulated protein kinase and phosphatase signaling that are associated with cardiovascular or other diseases could be therapeutically targeted. Successful clinical implementation of these compounds has been demonstrated for the treatment of various cancer types, with a focus on inhibition of Bruton’s tyrosine kinase and epidermal growth factor receptor (for a review see [212]). As an alternative to interfering with ATP binding, a protein kinase inhibitor could be designed to prevent substrate binding or dimerization as exemplified by the fluoromethylketone peptide PKI14-22, an experimental PKA inhibitor that binds to Cys199 and thus inhibits the PKA catalytic subunit [213] or the ERK-dimerization inhibitory peptide that attenuates cardiac hypertrophy and cancer progression [214]. A promising strategy that selectively modulates state-specific signaling molecules was shown for the protein tyrosine phosphatase 1B (PTP1B) that plays an important role in the regulation of insulin and leptin signaling. PTP1B is inactive in its reversibly oxidized form, which is paralleled by conformational rearrangement of the catalytic site. Using conformation-sensor antibodies, the oxidized state can be stabilized consequently switching phosphatase activity off [215].

In order to apply therapeutic strategies to modulate the highly important PKA signaling network either by global pro-oxidative intervention or selective cysteine modification, detailed research is invaluable to better understand the regulatory processes underlying oxidation-mediated PKA, PP1 and PP2A activity and to determine the specific contributions of each of the enzymes to oxidant-induced changes in protein phosphorylation and cardiac contractility.

## 11. Conclusions

The physiological role of ROS in the modulation of the PKA signaling pathway to ascertain unperturbed physiological functions is a relatively new concept. It took over 40 years to pinpoint the inducible character of PKA-RI oxidation that not merely is a purification artefact or a constitutive modification, but occurs dynamically inside the reducing cellular environment upon controlled ROS production. Thus far, the exact signaling scenario that impacts for example on excitation-contraction coupling—whether RI oxidation activates the kinase, how and if it triggers the cAMP-independent release of the catalytic subunits or whether alteration in AKAP affinity represents the main clue of RI interdisulfide formation—remains the topic of intense future investigations. In addition, oxidant susceptibility of the other signaling molecules that are players in the PKA-signalosome, namely the phosphatases PP2A and PP1, the respective AKAPs and even the catalytic subunit itself complicates the picture. When and whether this knowledge on reversible oxidation of the PKA signalosome will (ever) result in an innovate therapeutic treatment strategy, still requires a long and arduous scientific effort to gain a deeper understanding involving the generation of sophisticated experimental models. But, nevertheless, the outcome might represent the right avenue to tread for significant benefit in the therapy of heart failure and other diseases involving the PKA signaling pathway in the future.
